# A Procedure for High Resolution Satellite Imagery Quality Assessment

**DOI:** 10.3390/s90503289

**Published:** 2009-05-05

**Authors:** Mattia Crespi, Laura De Vendictis

**Affiliations:** 1 DITS, Area di Geodesia e Geomatica - Sapienza Università di Roma - via Eudossiana 18 - 00184 Rome, Italy; 2 Telespazio S.p.A. - via Tiburtina 956 - 00156 Rome, Italy; E-mail: guest517.devendictis@telespazio.com (L.D.)

**Keywords:** High Resolution Satellite Imagery quality, radiometric analysis, noise, Modulation Transfer Function, actual resolution

## Abstract

Data products generated from High Resolution Satellite Imagery (HRSI) are routinely evaluated during the so-called in-orbit test period, in order to verify if their quality fits the desired features and, if necessary, to obtain the image correction parameters to be used at the ground processing center. Nevertheless, it is often useful to have tools to evaluate image quality also at the final user level. Image quality is defined by some parameters, such as the radiometric resolution and its accuracy, represented by the noise level, and the geometric resolution and sharpness, described by the Modulation Transfer Function (MTF). This paper proposes a procedure to evaluate these image quality parameters; the procedure was implemented in a suitable software and tested on high resolution imagery acquired by the QuickBird, WorldView-1 and Cartosat-1 satellites.

## 1. Introduction

The second generation of high resolution satellites actually allows the acquisition of both panchromatic - with resolution from 0.5 m to 2.5 m - and multispectral imagery - with resolution from 2.4 m to 5.0 m, that offer a suitable alternative to aerial photogrammetric data for cartographic purposes as the updating and production maps at 1:5000 scale or lower, and the generation of Digital Surface Models (DSM).

Although HRSI still cannot replace aerial photos that provide resolutions as high as 0.1 ÷ 0.2 m, satellite remote sensing offers some advantages because it allows an easier acquisition of the same area at regular intervals, which is useful for monitoring natural or technological phenomena evolving in time and to easily obtain images of areas where it may be difficult to carry out photogrammetric flights (e.g. developing countries).

Within the chain from image sensing to the final value-added product, the quality of the images obviously plays a crucial role. The radiometry of an image is satisfactory when the relationship between the ground reflectance of the target and the grey level of the pixel on the image are correct. Nowadays, most of the linear array sensors have the ability to provide more than 8-bit/pixel digital images, meaning better radiometric performance (e.g. higher dynamic range and signal-to-noise ratio) with respect to traditional scanned 8-bit/pixel images. Nevertheless, we still have to consider some radiometric problems such as the variations in the sensor view angle, the sun angle and shadowing, the image noise that can influence the image matching algorithms and the image unsharpness due to CCD line jitter, kappa jitter and motion blur, and deficiencies of the lens system [1]. Image quality is represented by several parameters as the radiometric resolution and its accuracy, represented by the noise level and the geometrical resolution and sharpness, described by the Modulation Transfer Function (MTF).

In this paper a procedure for the estimation of the noise level and of the MTF of high resolution satellite imagery is presented. The methodology has been implemented in an appropriate software and tested on several images acquired by the QuickBird, WorldView-1 and Cartosat-1 satellites.

## 2. Image Noise Analysis

Several image-based methods such as the Homogeneous Area (HA) [2], Nearly Homogeneous Area (NHA) [3] and Geostatistical (GS) [4] ones were developed for the signal-to-noise ratio (SNR) estimation for remotely sensed images. Other researchers [5] consider that the noise may be dependent on land cover type, then they propose the modified GS method that estimates the SNR as a function of the both wavelength and land-cover types.

Here the noise level is evaluated through the standard deviation of the Digital Number (DN) in homogeneous and inhomogeneous areas according to the method proposed in [1,6]. Usually the image noise is estimated using the standard deviation of the DN in homogeneous areas where one type surface's pixels should have the same DN; in any case homogeneous areas are not really representative of a standard acquisition and, moreover, the use of inhomogeneous areas allows an analysis of the noise variation as a function of intensity.

Nevertheless, if inhomogeneous areas are considered, it has to be taken into account on the entire image that the DN differences can be due both to the different texture and to the noise; then the objective is to separate the noise from the effect of texture variations.

**Figure 1.**
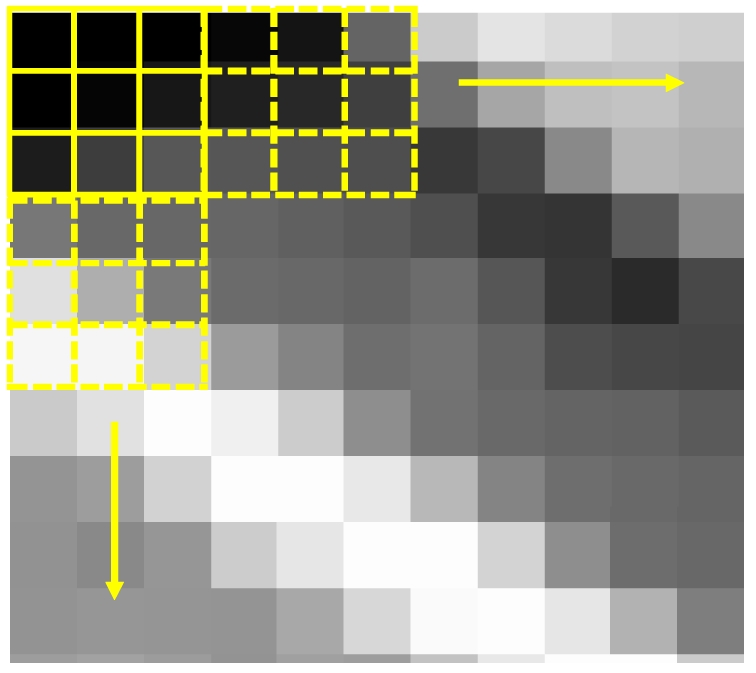
3 × 3 pixels window moving within the area by a 3 pixel step.

In order to achieve this aim a small squared window n × n pixels (for example, 3 × 3 pixels) wide is moved within the area by a 3 pixel step and the DN mean (M_w_) and the standard deviation (σ_w_) is calculated for each window (Figure 1).

The total DN range is divided into classes and the standard deviations are assigned to a class according to the mean DN of each window. Moreover, at this stage, each class contains all the standard deviations pertaining to those windows whose mean DN is within the DN limits of the class. It is reasonable that the lowest standard deviation are mainly due to the noise, whereas the other and for sure the highest are due to texture variations. Therefore in each class, the standard deviations are sorted, and the noise is estimated as the mean of the 5% smallest standard deviations; the noise is estimated for a class only if the 5% sample number is sufficiently large (e.g. > 100) [1,6]. Moreover, for a robust noise level estimation, instead of using just the mean of the 5% smallest standard deviation, the relative value R as an indicator for the signal-to-noise ratio [1] has to be used:

(1) $$\text{R}={(\text{DN}}_{\text{max}}-{\text{DN}}_{\text{min}}{)/\sigma }_{\text{s}}$$

where, σ_S_ is the noise estimation previously estimated for each class and DN_max_ and DN_min_ are the maximum and minimum DN of the image (dynamic range); they are computed from the whole image by excluding 0.5% pixels at the left and right sides of the image histogram. With this method the value of the image noise is normalized with respect to the dynamic range of the image and images with lower noise level should have a higher value of R.

It has to be noted that the 5% threshold is considered a reasonable choice and it may be slightly variable. A possible criterion to point out the threshold could be based on the computation of the mean σ_w_ at different percentiles and its interpolation with a smooth function: the threshold could be chosen where the second derivative of the interpolating function becomes significant.

**Figure 2.**
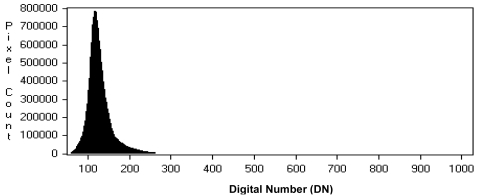
Cartosat-1 image DN histogram.

Regarding the DN classification, it is necessary to underline that, nowadays, even if most of the linear array sensors should be able to provide more than 8-bit/pixel digital images, the original digital images usually show poor image contrast, i.e. the peak of their histogram is usually located in the lower DN, with the right part of the histogram decreasing smoothly and slowly towards the higher values. Usually most of the data (> 80%) are contained in an interval of 255 DN, as shown in Figure 2 for Cartosat-1 imagery, collected in 10-bits format (1,024 available DN).

For this reason, in order to define meaningful classes when performing the signal-to-noise ratio analysis, the whole imagery gray interval has been divided into narrower classes (32 grey levels wide) for the DN range including most of the data and in wider classes (255 grey levels wide) in the other parts of the histogram.

Moreover, it is obvious that the noise level is dependent on the ground sampling distance (GSD): a larger GSD causes a higher σ_w_ and lower R, since it is much likely to find texture variation also if a small (3 × 3) window is considered. For example, if WorldView-1 and Cartosat-1 are compared, a (3 × 3) window is 2.25 m^2^ for WorldView-1 and 56.25 m^2^ for Cartosat-1. Window size could be larger for the sensors that have a GSD < 1 m; this aspect is of ongoing investigation. Anyway, the choice of a 3 × 3 window size represents a reasonable compromise between the needs of selecting not only homogeneous areas, which would not be sufficiently representative, and of not including remarkable texture variations.

The methodology has been previously tested on a simulated image, suitably generated to represent a 10-bit image with a DN histogram similar to a Cartosat-1 one, with 90% of data within the 0 – 255 DN interval. This image includes both areas with constant DN and areas where DN changes linearly along one direction (linear ramp).

**Figure 3.**
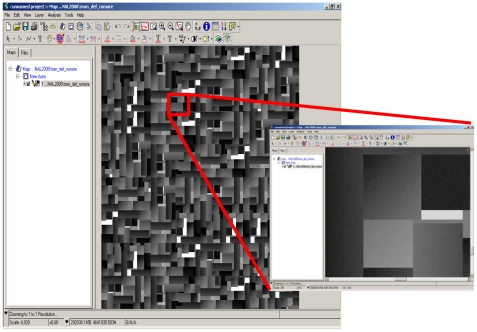
Simulated noisy image and a zoomed portion.

**Figure 4.**
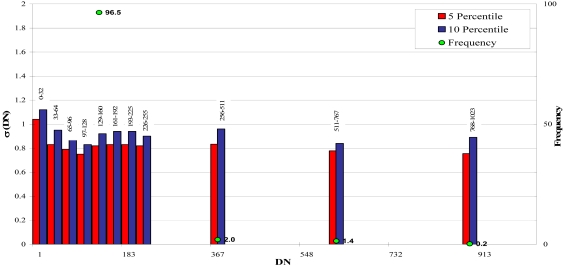
Simulated image noise level (σ) estimation at 5 and 10 percentile.

An artificial noise (standard deviation σ=1, mean M = 0) was added to the simulated image (Figure 3). For the noise estimation the whole imagery bit interval has been divided in narrower classes (32 grey levels wide) within the 0 – 255 DN interval and in wider classes (255 grey levels wide) in the other part of the histogram (DN within 256 – 1,024 interval). In each class, the standard deviations are sorted, and the noise is estimated as the mean of both the 5% and the 10% smallest ones, in order to evaluate the influence of the percentile (Figure 4). The results show that the proposed methodology is able to recover correctly enough the added noise, with a slightly underestimation if 5 percentile is considered. Anyway, we choose the lower percentile since real imagery are likely to be more textured, what increases the overall and the 5% lower standard deviation of the 3 × 3 moving window.

## 3. Post-Flight Modulation Transfer Function (MTF) Analysis

The Modulation Transfer Function (MTF) of an imaging system describes the transfer of an input in spatial frequency domain. It is well known that MTF is a useful tool to describe the sharpness of an imaging system. Most of the time, the MTF characteristics are measured before the launch; however, they may change due to the vibration during the launch or some change in material properties in time. For that reason, for on-orbit MTF determination it is necessary to have an up-to-date performance assessment of spaceborne sensors. Multiple methods have been proposed for determining the MTF of remote sensing systems in-orbit. Some of them are based on comparing test images to other images whose MTF is already known [7]. Other strategies for evaluating MTF, such as the periodic targets and spotlight methods, the bi-resolution method or the use of a neural network [8], are summarized in [9]. Most of the procedures for determining MTF use specific artificial or natural targets on the ground for estimating the Edge Spread Function (ESF) and hence the Line Spread Function (LSF) and MTF [10,11].

**Figure 5.**
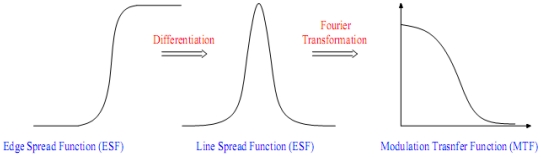
Explanation of the edge MTF estimation method.

According to the “Edge Method” proposed by Choi, a software package was developed to determine the MTF for high resolution satellite sensors. We started from this method because it is easy to implement and it is supported by tests on real images and independent data from the companies owning and managing the satellites. After the selection of some suitable edges in the image, at first the algorithm determines edge locations with sub-pixel accuracy; under the assumption that the chosen edges are on a straight line, the alignment of all edge locations is estimated with a least squares fitting technique. The edge profiles, which are centered at each edge pixel and have the direction perpendicular to the edge, are interpolated with cubic spline functions. These cubic spline functions, differently from Choi, are averaged and interpolated with an analytical function in order to obtain an empirical Edge Spread Function (ESF). The ESF is then differentiated to obtain the Line Spread Function (LSF). Finally the LSF is Fourier-transformed and normalized to obtain the corresponding MTF (Figure 5).

Finally, after the Fourier transformation, the computed MTF is scaled in the frequency axis in order to represent the calculated MTF in terms of the Nyquist frequency of the image. In addition, the Full Width at Half Maximum (FWHM) value is also computed from the estimated LSF. The details of the procedure are described in the following sections.

### 3.1. Edge selection

**Figure 6.**
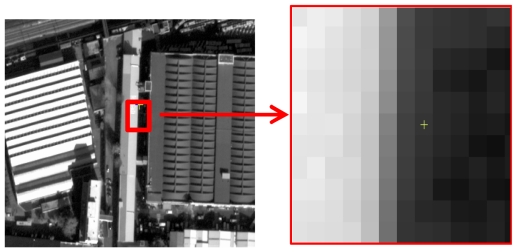
Edge example.

The initial task of the Edge Method is the identification of target edges useful for the analysis. Edges should show a blurred line edge between two relatively uniform regions of different intensity. The selected natural edges can be the separation line between the two layers of a roof, two fields with different cultivation, a roof edge and the ground, a road border and so on (Figure 6).

Edge targets that are constructed specifically for MTF measurements are generally preferred for MTF analysis; usually big tarps are used and they should be oriented near the principal along scan or across scan axes.

Another important parameter for the edge detection is the length of the edge profiles that should be long enough for reliably estimating the ESF and then LSF, but not too much long to avoid the possibility to introduce image noise effects. The number suggested by [1] with aerial images is of about 16 pixel. About high resolution satellite images, with a GSD > 0.5 m, since it is not always possible to use specially made targets, various natural and man-made almost linear structures with high-contrast are tried as imaging targets and often the edge profiles are not long more than 10 pixels.

Finally, edges must be well distributed and oriented near the principal along scan or across scan axes to take in account two different aspects: edges oriented perpendicular to the flight direction may be degraded by image motion or uncompensated vibrations such as the CCD line jitters and kappa jitters, while edges at the image border may be modified by lens aberrations.

### 3.2. Edge detection and least-square fitting line

**Figure 7.**
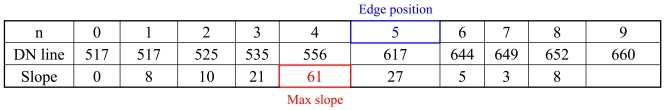
Edge position.

**Figure 8.**
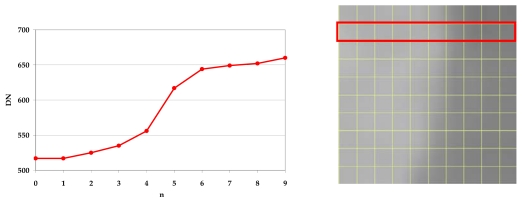
Profile of a line.

**Figure 9.**
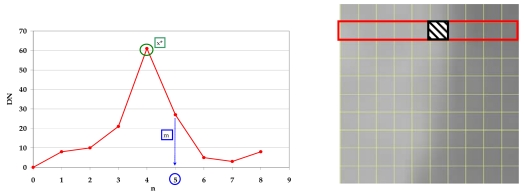
Differentiation and edge position estimation.

To establish the exact edge location line-by-line the DN values for each line were used; to detect the pixel m* where is approximately located the edge it is necessary to calculate the maximum slope by a simple discrete differentiation of the DNs (Figures 7, 8 and 9):

(2) Slope(x)=(DN[x+1]−DN[x])

where x = 0, …., (x_max_-2) is the pixel position of each line and x_max_= columns number.

Then it is possible to find m* by:

(3) $$\text{if}\;{\text{S}}_{\max}=\text{Max Slope}\;\left({\text{x}}^{*}\right)\Rightarrow {\text{m}}^{*}=\text{x}*+1$$

Now it's possible to estimate the sub-pixel edge points by fitting a cubic polynomial function to the edge data using seven values centered on the maximum slope point (4):

(4) $$\text{y}={\text{a}}_{1}x^{3}+{\text{a}}_{2}{\text{x}}^{2}+{\text{a}}_{3}\text{x}+{\text{a}}_{4}$$

Choi suggests to use only four values around the maximum slope point, but in this case it can happen that the sub pixel edge point results are not coherent with m*.

**Figure 10.**
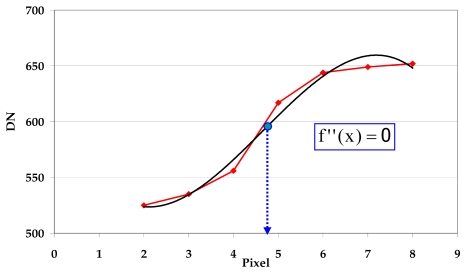
Sub-pixel location estimation.

After the polynomial coefficients estimation it is possible to assume the sub-pixel edge location x at the zero crossing location of the second derivative of the cubic polynomial equation (5) (Figure 10).


(5) $$\frac{\partial {\text{y}}^{\prime \prime }}{\partial \text{x}}=0\Rightarrow \text{x}=-\frac{2{\text{a}}_{2}}{6{\text{a}}_{1}}$$

**Figure 11.**
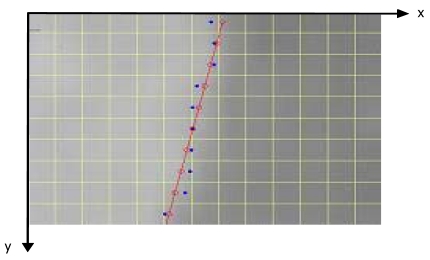
Fitted edge.

Since we assume that the edge is rectilinear and that any deviations from a straight line edge may represent errors in the geometry of the image, adopting a least squares approach the sub-pixel edge locations are fitted by a straight line x (6) (Figure 11):

(6) x=a⋅y+b

where x = 0, …, (x_max_-1) with x_max_= columns number; y = 0, …, (y_max_-1) with y_max_=rows number

### 3.3. Edge Spread Function (ESF)

**Figure 12.**
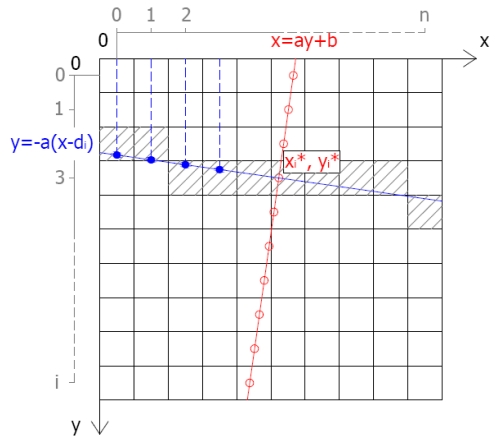
Line perpendicular to the edge.

The fitted edge is identified as described above. For each row i different straight lines x (7) are then constructed perpendicular to the edge and crossing the edge pixel in position ${\text{x}}_{\text{i}}^{*}$, ${\text{y}}_{\text{i}}^{*}$ (Figure 12):

(7) $$\text{x}=-\frac{1}{\text{a}}\text{y}+{\text{d}}_{\text{i}}$$

where

(8) $${\text{d}}_{\text{i}}={\text{x}}_{\text{i}}^{*}+\frac{1}{\text{a}}{\text{y}}_{\text{i}}^{*}$$

with

(9) $${\text{y}}_{\text{i}}^{*}=\text{i}+0.5$$

(10) $${\text{x}}_{\text{i}}^{*}=\text{a}\cdot {\text{y}}_{\text{i}}^{*}+\text{b}$$

where i = 0, 1,…, (i_max_-1) with i_max_ = rows number.

Then it is possible to find the row i (in correspondence of the centre pixel of each column) where the perpendicular line passes and the relative DN value:

(11) i=y(x)truncated

with

(12) $$\text{y}\left(\text{x}\right)=-\text{a}\cdot \left(\text{x}-{\text{d}}_{\text{i}}\right)$$

where x = [(0, 1, …, x_max_-1) + 0.5] with x_max_ = columns number.

This process is then repeated for each row of image data along the edge.

The DN values of the pixels along the perpendicular lines are interpolated with cubic splines [12,13]. It means that if N data (DN) are available, whose abscissas are ordered in increasing way (13):

(13) $${\text{x}}_{0}<{\text{x}}_{1}<\ldots <{\text{x}}_{\text{N}-1}$$

the goal is to estimate the S(x) function formed by N-1 cubic polynomial pieces S_k_(x):

(14) $${\text{S}}_{\text{k}}(\text{x})={\text{a}}_{\text{k}}{\left({\text{x}}_{\text{k}}\right)}^{3}+{\text{b}}_{\text{k}}{\left({\text{x}}_{\text{k}}\right)}^{2}+{\text{c}}_{\text{k}}({\text{x}}_{\text{k}})+{\text{d}}_{\text{k}}$$

for k = 0, …, N-1.

Then S(x) function will be:

(15) $$\text{S}\left(\text{x}\right)=\{\begin{array}{rr} {\text{S}}_{0}\left(\text{x}\right) & {\text{x}}_{\text{o}}<\text{x}<{\text{x}}_{1}\\ {\text{S}}_{1}\left(\text{x}\right) & {\text{x}}_{1}<\text{x}<{\text{x}}_{2}\\ \ldots & \ldots \\ {\text{S}}_{\text{N}-1}\left(\text{x}\right) & {\text{x}}_{\text{N}-2}<\text{x}<{\text{x}}_{\text{N}-1} \end{array}$$

In the ends of each intervals, sayings nodes (knots), conditions (I, II) of continuity of the function S(x) and of its derivatives of order first and second (III, IV) are to be satisfied (16)

(16) $$\{\begin{array}{ccc} \text{I} & {\text{S}}_{\text{k}}\left({\text{x}}_{\text{k}}\right)={\text{y}}_{\text{k}} & \text{k}=1,2,\ldots \text{N}-2\\ \text{II} & {\text{S}}_{\text{k}-1}\left({\text{x}}_{\text{k}}\right)={\text{y}}_{\text{k}} & \text{k}=1,2,\ldots \text{N}-2\\ \text{III} & {{\text{S}}^{\prime }}_{\text{k}-1}\left({\text{x}}_{\text{k}}\right)={{\text{S}}^{\prime }}_{\text{k}}\left({\text{x}}_{\text{k}}\right) & \text{k}=1,2,\ldots \text{N}-2\\ \text{IV} & {{\text{S}}^{\prime \prime }}_{\text{k}-1}\left({\text{x}}_{\text{k}}\right)={{\text{S}}^{\prime \prime }}_{\text{k}}\left({\text{x}}_{\text{k}}\right) & \text{k}=1,2,\ldots \text{N}-2 \end{array}$$

It has to be noted that for each polynomial piece k there are four unknown parameters (a_k_, b_k_, c_k_, d_k_) to be estimated, therefore, for (N-1) intervals 4(N-1) parameters have to be determined. Conditions (I, II, III, IV) supply 4(N-2) equations only, so that it is mandatory to add four additional conditions; conditions V and VI impose first and last values of the function:

(17) $$\{\begin{array}{ccc} \text{V} & \;{\text{S}}_{\text{k}}\left({\text{x}}_{\text{k}}\right)=0 & \;k=0\\ \text{VI} & \;{\text{S}}_{\text{k}}\left({\text{x}}_{\text{k}}\right)={\text{y}}_{\text{k}} & \;\text{k}=\text{N}-1 \end{array}$$

By the last two conditions VII and VIII, known as not-a-knot condition, the polynomial in the first two (and the last two) intervals are forced to being equal

(18) $$\{\begin{array}{cc} \text{VII} & {{\text{S}}^{\prime \prime \prime }}_{0}\left(x_{1}\right)={{\text{S}}^{\prime \prime \prime }}_{1}\left({\text{x}}_{1}\right)\\ \text{VIII} & {{\text{S}}^{\prime \prime \prime }}_{\text{N}-2}\left({\text{x}}_{\text{N}-2}\right)={{\text{S}}^{\prime \prime \prime }}_{\text{N}-1}\left({\text{x}}_{\text{N}-2}\right) \end{array}$$

After having estimated the S(x) function twenty values between two actual data points to build a pseudo-continuous line were introduced.

**Figure 13.**
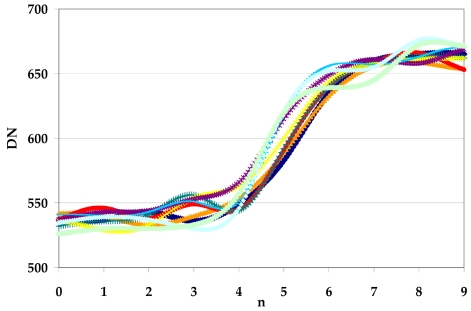
Splines interpolating perpendicular lines.

**Figure 14.**
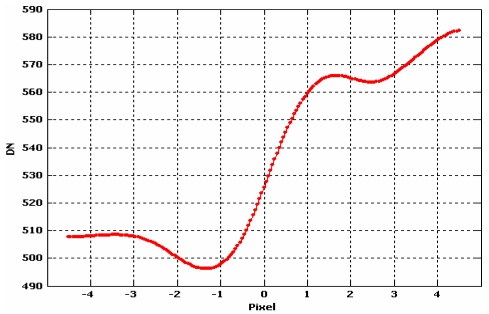
Edge Spread Function.

This procedure is repeated for each perpendicular line and finally, all the estimated cubic splines were used to get one averaged function that could represent the empirical Edge Spread Function (Figures 13 and 14).

### 3.4. Line Spread Function (LSF)

**Figure 15.**
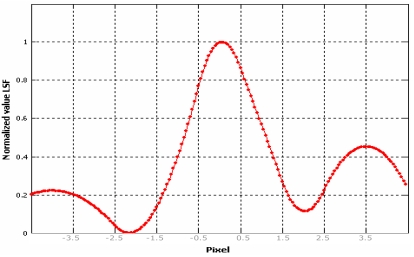
Line Spread Function.

Once the Edge Spread Function has been determined (Figure 11), the Line Spread Function (LSF) of the system should be computed by a simple discrete differentiation of the ESF (Figure 15):

(19) LSF(n)=ESF(n)−ESF(n−1)

**Figure 16.**
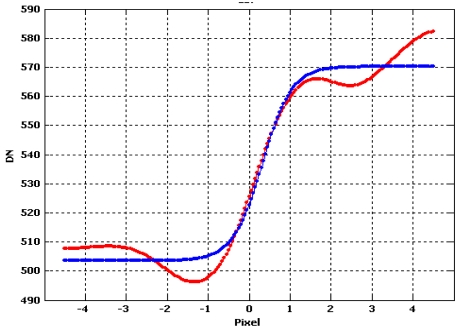
Empirical Edge Spread Function in blue color.

Choi [10] suggests to trim the LSF profile to reduce the noise present in the uniform areas on either side of the edge but, in our experience, this choice could not be always be appropriate to solve the problem, especially when natural edges are used, since they are usually more noisy. The presence of irregularities (lobes) at the sides of the LSF leads to an underestimation the Modulation Transfer Function. For this reasons we propose to interpolate the averaged function by function (20); it is a simple analytical function that models quite well the Edge Spread Function (Figure 14), suppressing the bad effect of possible lobes at the sides of the derived LSF (Figure 16):

(20) $$\text{y}=\frac{\text{a}}{1+{\text{e}}^{\left(\frac{\text{x}-\text{b}}{\text{c}}\right)}}+\text{d}$$

**Figure 17.**
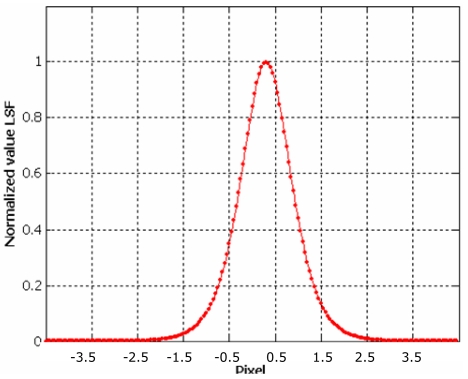
Line Spread Function from the empirical Edge Spread Function.

The LSF is calculated again according to equation (19); then LSF is normalized and trimmed in the interval (-5, 5) pixels with respect to the central value (Figure 17).

The inversion of the steps of the procedure, at first fitting each perpendicular line interpolated with splines with function (20) and then obtaining the ESF by averaging these models, could provide slightly different results, although the procedure is quite robust. Anyway, it would be interesting to investigate the possibility of eliminating some profiles of perpendicular lines, interpolated with splines, which results outliers with respect to their median. This analysis is in progress.

### 3.5. Modulation Transfer Function

**Figure 18.**
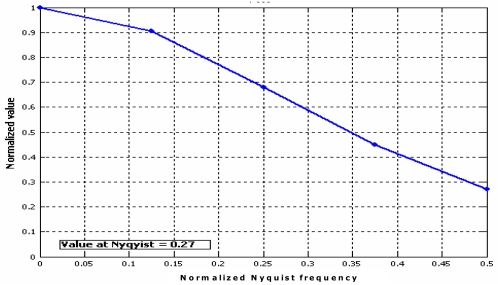
MTF at Nyquist frequency.

The MTF is obtained by Fourier transforming the LSF and scaling it in the frequency axis, in order to represent the MTF at the Nyquist frequency. The location of the Nyquist frequency was found using the equation (21), taking in account the data set size and the spline resolution, which was 0.05 pixels (Figure 18):

(21) Nyquist frequency=(whole data size×resolution)/2+1

### 3.6. Full-Width at Half-Maximum (FWHM)

**Figure 19.**
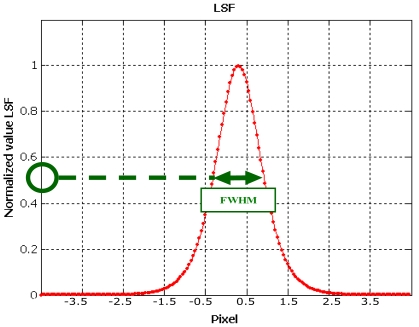
Full Width at Half Maximum.

The Full Width at Half Maximum (FWHM) value of the normalized LSF was also calculated as the difference between the abscissas at 0.5 (Figure 19). This index can be considered the actual image resolution.

**Figure 20.**
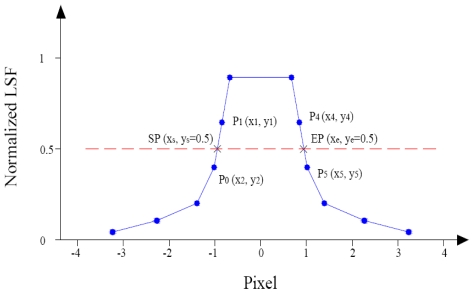
Method to estimate FWHM.

The FWHM was calculated by measuring (Figure 20) distance between the starting point and ending point individuated by a linear interpolation (22, 23):

(22) $$\{\begin{array}{c} {\text{x}}_{\text{s}}=\left(0.5-{\text{y}}_{1}\right)\cdot \frac{{\text{x}}_{2}-{\text{x}}_{1}}{{\text{y}}_{2}-{\text{y}}_{1}}+{\text{x}}_{1}\\ {\text{x}}_{\text{e}}=\left(0.5-{\text{y}}_{4}\right)\cdot \frac{{\text{x}}_{5}-{\text{x}}_{4}}{{\text{y}}_{5}-{\text{y}}_{4}}+{\text{x}}_{4} \end{array}$$

(23) $$\text{FWHM}={\text{X}}_{\text{e}}-{\text{X}}_{\text{s}}$$

## 4. Analysis of Results

The methodology described in the previous paragraphs has been tested on some high resolution imagery acquired by the QuickBird, WorldView-1and Cartosat-1 satellites. The QuickBird satellite, launched on 2001, allows acquisition of panchromatic imagery at a resolution of 61 cm at nadir. The WorldView-1 satellite, launched on 2007, actually collects the highest resolution commercial imagery of Earth; in fact it is the only half-meter resolution commercial imaging satellite, capable of collecting images with 50-centimeter panchromatic resolution. The Cartosat-1 sensor is dedicated to stereo viewing, carrying on board two pushbroom cameras, namely Aft and Fore, tilted in an along track direction by -5° and +26°, that provide stereoscopic imagery (BandA and BandF) in the same pass with a ground resolution of 2.5 m.

### 4.1. Data description

The dataset available for the experimentation consists of 10 panchromatic images.

- Four QuickBird
- Two WorldView-1
- Four Cartosat-1

The QuickBird dataset consists of four images:

- Two images of Standard OrthoReady type belonging to a pseudo-stereopair acquired over Colli Albani area (Rome, Italy)
- Two images derived from an acquisition over Rome (Italy) at two different levels of processing: Basic and Standard OrthoReady

**Figure 21.**
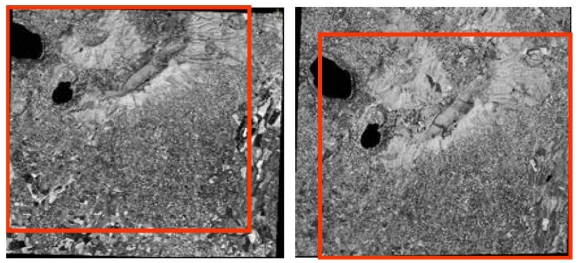
(a) QB_CA_StdOr_Right image. (b) QB_CA_StdOr_Left image. In red the overlap area.

The two images of the pseudo-stereopair (Standard Orthoready type), relative to the area of Colli Albani, south of Rome (Central Italy), were acquired from different viewing angles during two different orbital tracks, with a cross-track overlapping of almost 70% (15 × 15 km^2^). They are referred in the following as QB_CA_StdOr_Right (Figure 21a) and QB_CA_StdOr_Left (Figure 21b) images, according to the satellite azimuth and elevation values.

The QB_CA_StdOr_Right image was acquired on 12^th^ August 2003 with a mean cross-track viewing angle of 10.4 degrees, and a mean resolution of 0.63 m; the QB_CA_StdOr_Left image was acquired on 6^th^ July 2004, with a mean cross-track angle of 17.7° and a mean resolution of 0.66 m.

**Figure 22.**
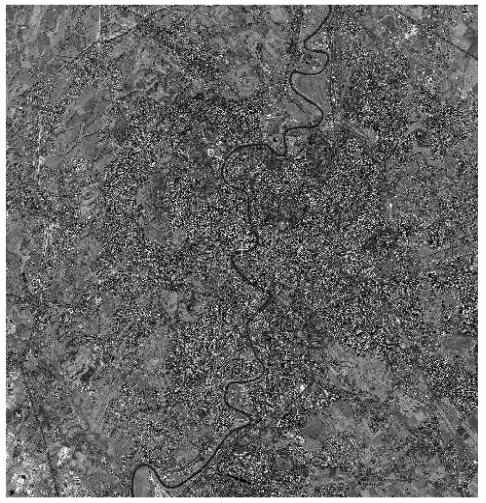
QuickBird Rome image.

The QuickBird image over Rome (Figure 22) was acquired on 5^th^ April 2005 with a mean cross-track angle of 2.1° at a nominal resolution of 61 cm. For the experimentation two different products – Basic and Standard OrthoReady – derived from two different levels of processing (Basic and Standard OrthoReady), were available. They are referred in the following as QB_RM_Basic and QB_RM_StdOr, according to the processing level.

Basic Imagery products are radiometrically corrected and sensor corrected, but not geometrically corrected nor mapped to a cartographic projection and ellipsoid. Image resolution varies between 61-centimeters (at nadir) to 72-centimeters (25° off-nadir look angle). Standard OrthoReady Imagery products are radiometrically corrected, sensor corrected, projected onto a surface parallel to the WGS84 ellipsoid and mapped to a cartographic projection. All Standard OrthoReady Imagery products have a uniform pixel spacing (in this case 60 cm) across the entire product.

**Figure 23.**
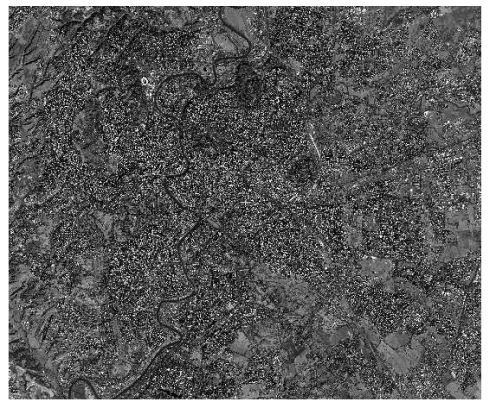
WorldView-1 Rome image.

The WorldView-1 image (Figure 23) was acquired over Rome on 15^th^ February 2008 with a mean cross-track angle of 6.5 degrees at a nominal resolution of 51 cm. Also in this case, two different products, Basic and Standard OrthoReady, derived from a different levels of processing, were available. The characteristics of the two level products are the same of QuickBird products. They are referred in the following as WV1_RM_Basic and WV1_RM_StdOr, according to the processing level.

**Figure 24.**
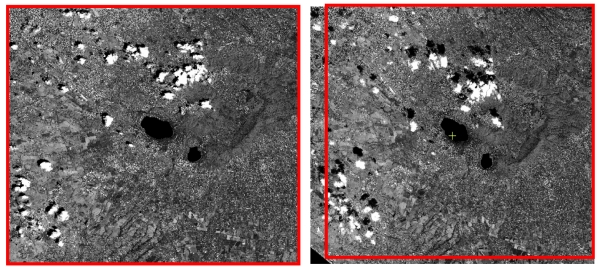
(a) CSAT1_CA_BandA.(b) CSAT1_CA_BandF. In red the overlap area.

**Figure 25.**
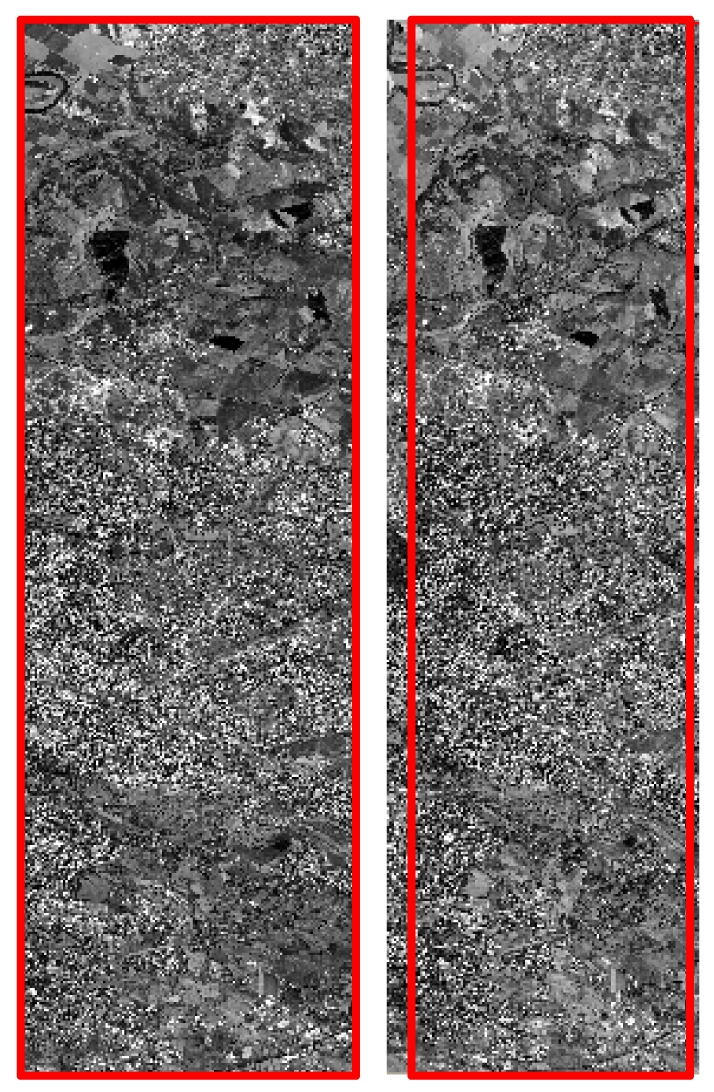
(a) CSAT1_RM_BandA.(b) CSAT1_RM_BandF. In red the overlap area.

The Cartosat -1 dataset consists of four images:

- Two stereo images (Figure 24a and b) acquired over Colli Albani area on 18^th^ July 2006 with a ground resolution of 2.5 m.
- Two stereo images (Figure 25a and b) acquired over Rome and suburbs on 8^th^ June 2005 with a ground resolution of 2.5 m.

The Colli Albani stereo scenes have an in-track overlap of almost 90% and each image has a size of 12,000 pixel – 12,000 pixels, with a ground resolution of 2.5 m. They are referred to in the following as CSAT1_CA_BandA (Figure 24a) and CSAT1_CA_BandF (Figure 24b) images, according to the satellite acquisition angles.

The stereopair over Rome is not a standard stereopair, since it was acquired just one month after the launch and this usually poses questions about their quality, since one month is usually not sufficient for the calibration and validation of a satellite sensor; moreover only 3,000 (on a total amount of 12,000) sensor detectors were active. They are referred in the following as CSAT1_RM_BandA (Figure 25a) and CSAT1_RM_BandF (Figure 25b) images, according to the satellite acquisition angles.

### 4.2. Signal-to-noise ratio estimation

The noise of the images was estimated according to the method described in paragraph 2, that quantifies the noise characteristics of the images using the standard deviation of the DN in non- homogeneous areas, allowing an analysis of the noise variation as a function of intensity. The QuickBird and WorldView-1 imagery are collected in 11-bits format (2,048 grey levels) but, even if the peak is less pronounced, the 80% of the DN vary between 256 and 511. The Cartosat-1 sensor provides images with 10 bit/pixel, that means 1,024 available grey levels, but the 99% of the DN vary between 0 and 255.

For this reason, in order to perform the signal-to-noise ratio analysis the whole imagery DN intervals have been divided in different classes: 32 grey levels wide for the DN range including most of the data and 255 grey levels wide in the other part of the histogram.

**Figure 26.**
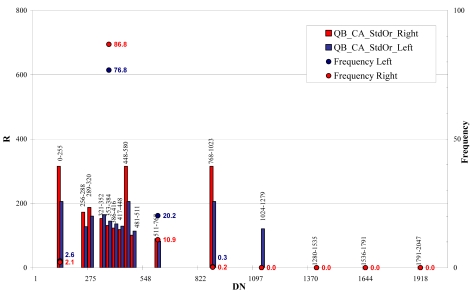
QB_CA signal-to-noise ratio (R) level estimation.

**Figure 27.**
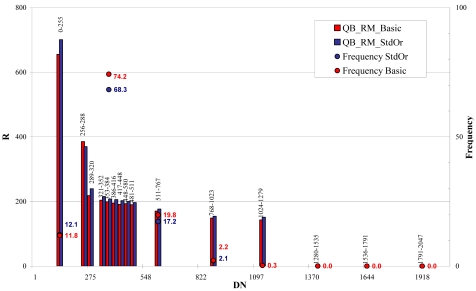
QB_RM signal-to-noise ratio (R) level estimation.

**Figure 28.**
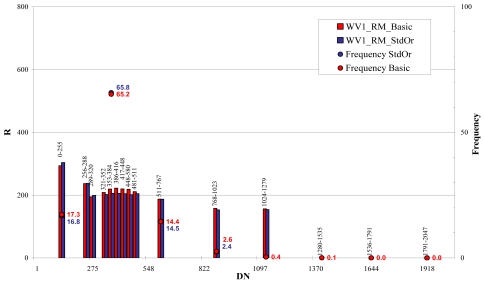
WV1_RM signal-to-noise ratio (R) level estimation.

For QuickBird images the whole bit interval (0 – 2,048) was divided in 15 classes, 32 grey levels wide between 256 and 511 bits and 255 grey levels wide, between 0 and 255 bits and between 511 and 2,048 bits (Figure 26 and 27). For WorldView-1 images the whole bit interval (0 – 2,048) was divided as for the QuickBird imagery (Figure 28).

The results showed, for both satellites, a practical independence between signal-to-noise ratio/DN in the most populated classes (80% of pixels)þ and a light correlation in the extreme classes (0 – 320 \ 512 – 1,279) where the noise increases with DN (QB_RM, Figure 27 and WV1_RM, Figure 28).

**Figure 29.**
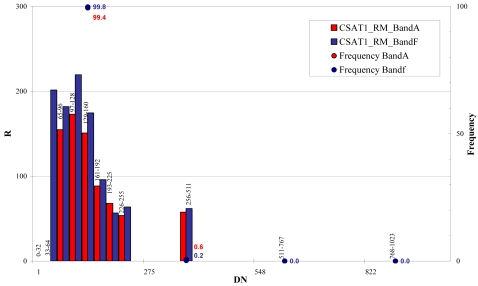
CSAT1_RM signal-to-noise ratio (R) level estimation.

**Figure 30.**
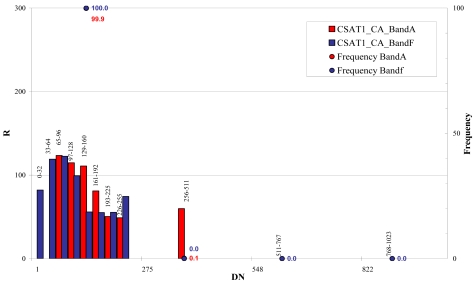
CSAT1_CA signal-to-noise ratio (R) level estimation.

The whole bit interval of the Cartosat-1 images (0 – 1,024) was divided in 11 classes, 32 grey levels wide between 0 and 255 bits and 255 grey levels wide, between 256 and 1,024 bits (Figure 29 and 30).

The noise computed for Cartosat-1 images indicates that noise is intensity dependent, in fact noise is increasing with increasing grey values. Some grey value ranges have no reliable standard estimation, due to the small number of samples (e.g. < 100). The higher noise level of these images is probably due larger ground pixel size of the images with respect to the QuickBird and WorldView-1 ones.

### 4.3. Modulation Transfer Function estimation

**Figure 31.**
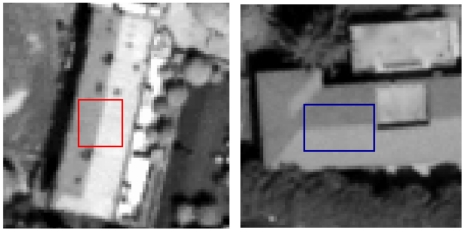
(a) along-track direction edges example. (b) across-track direction edges example.

The MTF was estimated using the “Edge Method” described in paragraph 3. Since it was not possible to use specially made targets, the first step consisted in detecting various natural and man-made almost linear structures, chosen to be a blurred line edges between two relatively uniform regions of differing intensity. In order to perform the MTF analysis both along-track and across-track direction, the chosen edges are orientated as much parallel to the along- or across-track direction as possible (Figure 31a, b). and are well distributed across the image.

On the available images several edges were selected:

- Forty five edges in both directions in the overlap area of the QuickBird Colli Albani pseudo-stereopair
- Twenty two edges in both directions in the overlap area of the QuickBird and WorldView-1 Rome images (both Basic and Standard OrthoReady)
- Twenty five edges in both directions on the overlap area of the Cartosat-1 Rome stereopair
- Twenty edges in both directions on the overlap area of the Cartosat-1 ColliAlbani stereopair

The MTF values at Nyquist frequency and the FWHM values were estimated for all the selected edges; the results were combined into average values for the along- and across-track direction (Table 1).

The results achieved for the QuickBird ColliAlbani images show that the MTF values and the FWHM values for both images are comparable and there seems to be no difference between the along and cross track directions. It has to be underlined that in this case the two images, Left and Right, were acquired with slightly different off-nadir and sun angles angle, but these parameters do not seem to affect remarkably MTF and FWHM values.

With respect to the QuickBird and WorldView-1 images, it can be noticed that the resolution of both satellites is better in the cross-track direction and that the Basic images display a slightly better quality. With regards to the Rome imagery, it has to be underlined that these images are almost nadiral and this aspect seems to influence positively the effective resolution in cross-track direction.

These results are in good agreement with [14], that assumes as reference for QuickBird a MTF value at the Nyquist frequency of approximately 0.17 along-track direction and of 0.21 cross-track direction.

It is to be noted that DigitalGlobe (company owning of QuickBird and WorldView-1 satellites) offers customers a Dynamic Radiometric Adjustment (DRA) option, which visually enhance imagery by performing color correction and contrast enhancement. In this experimentation all images are “DRA off”; the DRA application on one hand increases the contrast, on the other probably increases the noise.

It could be interesting to investigate the influence of the resampling option of Digital Globe products: 4 × 4 cubic convolution, 2 × 2 bilinear, nearest neighbor and MTF kernel. All the products available for the experimentation have a 4 × 4 cubic convolution resampling (CC); probably, adopting as resampling an MTF kernel can improve the results [15].

The results achieved for the Cartosat-1 images show that the MTF values for the along-track direction are always larger than those for the cross-track direction; moreover, the FWHM values for the BANDF image, that is acquired with an off-nadir angle of 26°, in the cross-track direction, are remarkably larger than those for the along-track direction. The Cartosat-1 images over Rome, acquired just one month after the launch, have not any kind of resampling; the stereopair over Colli Albani area have a cubic convolution resampling (CC), but it seems not to influence the results.

## 4. Conclusions

This paper proposes a complete methodology to assess the radiometric quality of high resolution satellite imagery estimating the noise level and analyzing the image sharpness, represented by the Modulation Transfer Function (MTF) and the actual resolution through FWHM. According to the method proposed in [6], the noise characteristics were analyzed in non homogeneous image regions, in order to evaluate the noise variation as a function of intensity. The results achieved show that for QuickBird and WorldView-1 images the noise does not seem to increase with the intensity, excluding a light correlation in the extreme parts of the grey level histograms where the noise increases with DN.

With regards to the Cartosat-1 images, the noise is intensity dependent since it grows with increasing grey values; it is larger compared to the QuickBird and WorldView-1 ones, probably due to the larger ground pixel size of the images.

The image sharpness is another important parameter for characterizing images quality. Image blur, which limits the visibility of details, can be objectively measured by the Modulation Transfer Function (MTF) that represents, in the spatial frequency domain and for a given direction, the effective image spatial resolution. To estimate the MTF, starting from the “Edge Method” proposed by [10], but introducing original upgrades in order to overcome noise problems close to the edege, the Edge Spread Function (ESF), hence the Line Spread Function (LSF) and finally the MTF, have been estimated using natural edges on the ground. In order to perform the MTF analysis in both along-track and cross-track direction, the edges have been chosen orientated as much parallel to the along- or cross-track direction as possible.

The results achieved show that, for Cartosat-1 images, the MTF values in the along-track direction are always larger than those for the cross-track direction, while the values obtained for QuickBird and WorldView-1 images, especially for nadiral images, the resolution is better in the cross-track direction and that the Basic images display a slightly better quality.

## Figures and Tables

**Table 1.** MTF and FWHM values vs. off-nadir angle, sun angle and resampling strategy.

**Image**	**Date**	**Off-Nadir**	**Sun Angle**	**Resampling**	**Edges cross-track**		**Edges along-track**	
					**MTF at Nyquist**	**FWHM (pixel)**	**MTF at Nyquist**	**FWHM (pixel)**
QB_CA_StdOr_Right	12/08/03	10.4°	57.5°	CC	0.22	1.50	0.19	1.59
QB_CA_StdOr_Left	06/07/04	17.7°	66.1°	CC	0.24	1.45	0.20	1.55
QB_RM_Basic	28/04/05	2.1°	60.2°	CC	0.25	1.31	0.19	1.52
QB_RM_StdOr	28/04/05	2.1°	60.2°	CC	0.23	1.37	0.17	1.60
WV1_RM_Basic	15/02/08	6.5°	32.2°	CC	0.23	1.40	0.17	1.60
WV1_RM_StdOr	15/02/08	6.5°	32.2°	CC	0.23	1.40	0.15	1.66
CSAT1_RM_BandA	08/06/05	-5.0°	68.5°	None	0.16	1.74	0.25	1.44
CSAT1_RM_BandF	08/06/05	26.0°	66.6°	None	0.07	2.52	0.16	1.88
CSAT1_CA_BandA	18/07/06	-5.0°	65.6°	CC	0.16	1.75	0.26	1.45
CSAT1_CA_BandF	18/07/06	26.0°	63.8°	CC	0.06	2.49	0.15	1.87
